# Development of a patient-centered peer-mentoring program for kidney transplant patients: a qualitative study

**DOI:** 10.3389/fpsyg.2025.1712921

**Published:** 2025-11-28

**Authors:** Martin Kumnig, Sheila Jowsey-Gregoire, Anna Gruber, Matthias M. Kofler, Elisa J. Gordon

**Affiliations:** 1 Department of Psychiatry, Psychotherapy, Psychosomatics and Medical Psychology, Center for Advanced Psychology in Plastic and Transplant Surgery, Medical University of Innsbruck, Innsbruck, Austria; 2 Department of Psychiatry and Psychology, Mayo Clinic Jacksonville, Jacksonville, FL, United States; 3 Faculty of Psychology and Sport Science, University of Innsbruck, Innsbruck, Austria

**Keywords:** end-stage kidney disease, dialysis, patient advocacy, psychosocial outcomes, quality of life, peer-mentoring, social support, health care navigation

## Abstract

**Background:**

End-stage kidney disease (ESKD) causes physical and mental challenges to affected patients that complicates their ability to manage pre-transplant medical care. Patients benefit from both education about their treatment options, and social support. Peers can address these needs if provided with suitable instruction and direction. This study identified patient-centered components of a peer-mentoring program for kidney transplant recipients based on the input from recipients and providers.

**Methods:**

We conducted semi-structured videoconferences with kidney transplant recipients (KTRs) to assess experiences of the pre-transplant evaluation process, post-transplant recovery, living with a kidney transplant, and preferences of a peer-mentoring program. Transcripts were analyzed using thematic analysis.

**Results:**

A total of 44 KTRs participated. Four themes emerged about integral components of a patient-centered peer-mentoring program: psychosocial challenges and adjustment after transplantation; navigating complex information and the effects of trusted peer experiences; the qualifications, role, and tasks of peer mentors; and barriers and facilitators to engaging in peer support. Participants recommended that peer mentors possess certain characteristics (e.g., communicational/psychological skills) and provide support (e.g., low-threshold sharing of information and experiences). Optimal peer-mentoring programs should adapt to patients' needs, address QOL concerns and transplant outcomes, and be easily available. Peer-mentor training should cover transplant-related medical information, the role of a peer mentor, communication training, and information about patient advocacy tasks.

**Conclusions:**

This study provides insights into developing and implementing a kidney peer-mentoring program. Future research should adapt currently existing peer-mentoring programs to accommodate these patient-centered qualities and evaluate accordingly.

## Introduction

The challenges that patients with end-stage kidney disease (ESKD) encounter are well known as they undergo renal replacement therapy, comprised of dialysis or kidney transplantation (Renz-Polster, 2012). While both options are physically and mentally demanding, kidney transplantation is considered the better option, as it provides lower mortality and higher quality of life (De Pasquale et al., 2020; Fletcher et al., 2022; Tonelli et al., 2011). Patients who struggle due to health issues, a lack of knowledge, or do not have a support system, may need additional support to overcome such challenges (Abbasinia et al., 2019) These challenges underscore the need for comprehensive educational and psychosocial support throughout the transplant trajectory (De Pasquale et al., 2020; Abbasinia et al., 2019; Lopez-Vargas et al., 2016).

Traditional transplant education primarily emphasizes biomedical aspects of care and may not fully address patients' lived experiences or psychosocial adjustment needs (Neyhart, 2008; Hart et al., 2019) As a result, patients commonly report information overload, anxiety, and difficulties in navigating medical systems or maintaining adherence (Ahsanuddin et al., 2015; Wright et al., 2001).

Peer mentoring—support offered by trained individuals who have undergone similar experiences—has emerged as an evidence-based approach that complements professional care by offering emotional, informational, and social support (Dennis, 2003; Sunderland et al., 2013). Peer-based interventions have demonstrated patient benefits including: improved self-efficacy, adherence, and psychosocial wellbeing in chronic illness contexts, including dialysis and transplantation (Taylor et al., 2016; Pomey et al., 2021; Longley et al., 2023). Peer mentors can facilitate the adequate and fair treatment for vulnerable patients (Abbasinia et al., 2019; Sullivan et al., 2012; Wells et al., 2016; Patzer and Larsen, 2019; Fischer et al., 2007; Phillips et al., 2019; Cervantes et al., 2020). Overall, research has shown that peer-based programs are associated with positive outcomes including improved adherence and self-management, increased knowledge, social support, completed transplant work-up (Haugen, 2019; Nishio-Lucar et al., 2020; Nishio Lucar et al., 2024), improvements in patient engagement, self-efficacy, psychological wellbeing (Longley et al., 2023; Husain et al., 2020), and increase equity in access to living donor kidney transplantation (Nishio-Lucar et al., 2020; Taha et al., 2022). Other peer-mentoring programs helped patients accept their diagnosis and choose a treatment modality (Jain et al., 2019).

While several factors have been identified that shape the development of a peer-mentoring program, limited research has elucidated how to develop a peer support training based on patient centered preferences and outcomes. In patient centered care, individuals' specific health needs and desired health outcomes are the driving force behind all health care decisions and quality measurements, empowering patients to become active participants in their care. Studies identified factors such as that a peer support program should be easily available (Elliott et al., 2023), be delivered via face-to-face interactions, phone calls, or online interactions, or a combination of different modalities (Pomey et al., 2021; Bennett et al., 2018), and involve the healthcare team (Pomey et al., 2021; Elliott et al., 2023; Bennett et al., 2018; Elliott et al., 2022; Trasolini et al., 2021; Wood et al., 2022). Thus, peer support works best in conjunction with the multidisciplinary care team. Ideally, training of peer supports is ongoing, facilitates networking, provides additional training opportunities, and promotes self-care for the peer supporters (Sunderland et al., 2013). Peer supporters do not need a professional education (Cervantes et al., 2020). An important feature of training peer volunteers is in providing medical information, not giving medical advice and differentiating their role from healthcare professionals (Sunderland et al., 2013; Pomey et al., 2021; St. Clair Russell et al., 2017). Peer training typically encompasses education on ESKD and kidney transplantation (Sunderland et al., 2013; Cervantes et al., 2020; St. Clair Russell et al., 2017).

Despite promising outcomes, the development of peer-mentoring programs in transplantation has generally been guided by institutional priorities (i.e., developing educational materials but not asking what are the patients' concrete needs in the transplant course) rather than patient perspectives. Therefore, a critical gap remains in understanding what kidney transplant recipients perceive as essential elements of a meaningful, patient-centered peer-mentoring program. Few studies have systematically examined how recipients conceptualize the role, content, and delivery of peer support tailored to their psychosocial and informational needs.

Due to the paucity of studies in this area (Longley et al., 2023), this study examined the psychosocial, informational, and support needs of kidney transplant recipients to inform the co-development of a patient-centered peer-mentoring program, and to ascertain critical insights into the experience and perspectives of patients (Tong et al., 2013). Specifically, we sought to assess: (1) the types of challenges that could be addressed through peer support, and (2) participants' preferences for the structure, content, and delivery of such a program.

## Methods

### Study design

This cross-sectional qualitative study was conducted at the Medical University Innsbruck from May 2023–September 2024. The study was approved by the ethics committee of the Medical University Innsbruck (vote number 1038/2023) and written informed consent was obtained after initial telephone contact. This qualitative research follows the Standards for Reporting Qualitative Research (SRQR) guideline (O'Brien et al., 2014).

### Inclusion criteria

Individuals were eligible for participation if they were age 18 years+, had received a kidney transplant at least 6 months prior to recruitment (including patients with repeated transplants; deceased donor and living kidney donor transplants) at one of the four Austrian transplant centers (Vienna, Innsbruck, Graz, and Linz), and are members of the ANÖ, spoke German, and could access the Internet. Recipients of multi-organ transplants (e.g., kidney-liver or kidney-pancreas) were excluded.

### Recruitment

We mailed/emailed a study invitation letter to the Arbeitsgemeinschaft Niere Österreich (ANÖ, Austrian Self-Help Association for Patients with ESKD) that disseminated the letter to all members. The ANÖ is a non-profit organization, member of the European Kidney Patients Federation (EKPF), receiving financial support by public revenues and donations, and works in close contact with all Austrian transplant programs. Interested patients replied to the emailed/mailed letter with contact information, providing basic information about themselves and their treatment course, to screen patients for eligibility. Thereafter, research staff called patients who gave written informed consent, and scheduled an interview date.

### Data collection

In-depth, semi-structured interviews were conducted about KTRs' experiences before and after undergoing kidney transplantation and their preferences for how a peer mentoring program could address those experiences. The open-ended questions allowed the respondents to discuss issues of significance without imposing the research team's own ideas and thus enable understanding of respondents' point of view (Gordon et al., 2009). The interview guide was comprised of 25 demographic and 30 open-ended questions and was developed by three researchers based on the literature (Bradley et al., 2007). The semi-structured interview format enabled a flexible and systematic approach so that the interviewer could ask questions that were relevant to each participant's own narrative. The interview guide included questions regarding the patients' experiences before (e.g., during the period of dialysis) and after kidney transplantation, patients' QOL/HRQOL as well as their post-transplant life (e.g., ability to go to work, individual perceived level of autonomy). Additionally, we assessed how patients perceived the informed consent process in the course of kidney transplantation, their recommendations and preferences on how to improve the level of support before and after kidney transplantation, and psychosocial issues they have encountered. We also assessed patients' perceptions of preferred characteristics and skills of peer mentors, and of factors to develop a peer support training program by asking patients about their experience-based recommendations and preferences. Interviews also included 25 demographic questions. We conducted interviews via videoconference meetings, using WebEx and Zoom or by telephone. Interviews lasted 60 min, were audio-recorded and transcribed verbatim.

### Qualitative analysis

Following the thematic synthesis methods approach (Thomas and Harden, 2008), transcripts were analyzed using thematic analysis assisted by qualitative analysis software (Atlas.ti software, version 9.1.7). Coding was guided by the research questions (Bradley et al., 2007). Thematic analysis entailed the identification of patterns or themes (Braun and Clarke, 2006). An initial coding scheme was generated based on reviewing ten transcripts by three independent reviewers. The feedback from the independent interrater process was used to develop the final coding scheme that consisted of 74 codes organized into four themes. Any discrepancies were resolved through discussion.

### Statistical analysis

The demographic data were analyzed using descriptive statistics (e.g., frequencies, SDs) with data analysis software SPSS, IBM Germany, München version 29.

## Results

### Participant characteristics

Forty-four kidney transplant recipients (KTRs) participated in this study. Participants had a mean age of 57.8 years (SD = 12.2), were predominantly male (68.2%) and married (56.8%), and nearly half had completed secondary education (47.7%). Most participants had undergone dialysis prior to transplantation (86.4%) and had received one transplant (79.5%). Additional sociodemographic and clinical characteristics are presented in Table 1.

**Table 1:** Sociodemographic and transplant-related characteristics of participants (*N* = 44).

**Sociodemographic characteristics**	
Age, M ± SD	57.82 ± 12.19
**Gender**, ***n*** **(%)**	
Men	30 (68.2)
Women	14 (31.8)
**Marital status**, ***n*** **(%)**	
Married	25 (56.8)
Permanent relationship (>1 year)	8 (18.2)
Single	7 (15.9)
Divorced/separated	3 (6.8)
Widowed	1 (2.3)
**Living situation**, ***n*** **(%)**	
With partner or family	32 (72.7)
Alone	10 (22.7)
Shared living space	2 (4.5)
**Education**, ***n*** **(%)**	
Matura or vocational secondary school	21 (47.7)
Compulsory school with apprenticeship or master training	13 (29.5)
University	10 (22.7)
**Employment status**, ***n*** **(%)**	
Retired	19 (43.2)
Full time employment	17 (38.6)
Part time employment	4 (9.1)
Other	3 (6.8)
Temporary pension/ongoing application	1 (2.3)
**Transplantation**	
Number of received kidney transplantations, *n* (%)	
1 kidney transplantation	35 (79.5)
2 kidney transplantations	9 (20.5)
Year of first transplantation, M ± SD	2,009.73 ± 10.57
Year of second transplantation, M ± SD	2,012.11 ± 8.52
Time span between first and second transplantation (in years), M ± SD	13.67 ± 8.65
**Donation type**, ***n*** **(%)**	
Post-mortem donation	30 (68.2)
Living donation	11 (25.0)
Both	3 (6.8)
**Donor**, ***n*** **(%)**	
Deceased donor	30 (68.2)
Spouse	5 (11.4)
Parent	3 (6.8)
Other family member	3 (6.8)
Friend	2 (4.5)
Child	1 (2.3)
**Waiting time in case of a postmortem donation (in years)**,	
**M** ±**SD**	
Waiting time for first transplant	2.56 ± 2.02
Waiting time for second transplant	2.41 ± 1.39
Dialysis	
**Dialysis obligation**, ***n*** **(%)**	
Yes	38 (86.4)
No	6 (13.6)
**Dialysis type**, ***n*** **(%)**	
Hemodialysis	19 (43.2)
Peritoneal dialysis	10 (22.7)
Both	9 (20.5)
No dialysis	6 (13.6)
**Duration of dialysis treatment (in years)**,	
**M** ±**SD**	
Before first transplant	2.15 ± 1.91
Before second transplant	2.75 ± 1.90
Changes in employment	
**Timeframe from transplantation to reentering the workforce**,	
***n*** **(%)**	
< 3 months	19 (43.2)
Not applicable	11 (25.0)
3–6 months	10 (22.7)
>12 months	3 (6.8)
7–12 months	1 (2.3)
**Change in profession after transplantation**, ***n*** **(%)**	
No	37 (84.1)
Yes	7 (15.9)

*N* = 44, M = mean, SD = standard deviation.

### Overview of themes

Four overarching themes covered participants' perspectives on transplant-related needs and preferences for a peer-mentoring program: (1) psychosocial challenges and adjustment after transplantation; (2) navigating complex information and the value of trusted peer experiences; (3) qualifications, roles, and matching of peer mentors; and (4) barriers and facilitators to engaging in peer support. See Table 2 for main themes and key subthemes.

**Table 2:** Themes and key subthemes identified in thematic analysis.

**Themes**	**Key subthemes**
1. Psychosocial challenges and adjustment after transplantation	•Improved physical functioning, psychological wellbeing, and quality of life after transplant •Persistent vulnerabilities: fluctuating emotional resilience, pain and fatigue, difficulty coping at critical recovery phases •Fear of graft rejection, heightened awareness of organ, challenges integrating graft into identity •Emotional burden toward living donors and concerns about burdening family •Social strain, isolation, changing family dynamics, need for assistance in daily life •Vocational reintegration: desire for independence vs. physical/psychological barriers and financial concerns •Peer mentors perceived as helpful for emotional normalization, coping support, and guidance in reintegration into family, social, and work roles
2. Navigating complex information and effects of trusted peer experiences	•Need for staged, comprehensible information across the transplant trajectory •Desire for practical, real-life insights on immunosuppression, recovery timelines, restrictions, and side effects •Early education on dialysis options, organ acceptance decisions (including marginal organs), donation types, and allocation processes •Interdisciplinary education (medical, psychological, nutritional, physical rehabilitation) •Peers facilitate translation of medical advice into everyday strategies and self-management •Value of multiple peer perspectives and early family involvement to reduce misunderstandings and anxiety •Preference for low-threshold educational resources (brochures, online platforms, group sessions, family-inclusive sessions)
3. Qualifications, roles, and matching of peer mentors	•Credible role models with extensive post-transplant experience and emotional stability •Strong communication skills, empathy, boundary-setting, and ability to separate personal experience from general information •Screening for psychological readiness and professionalism •Matching based on age, gender, treatment modality, language, education, and geography to enhance comfort discussing sensitive topics •Supportive companion role, not replacing healthcare professionals; clear scope boundaries •Flexibility to change mentor if fit is insufficient •Option for mentor presence during clinical visits (e.g., in waiting areas) when desired
4. Barriers and facilitators to engaging in peer support	•Topics needing targeted support: body image, sexuality and intimacy, fertility, occupational reintegration, disability, disclosure, lifestyle adjustments •Early introduction to peer support; variable preferences for program duration •Hybrid delivery preferred: in-person for trust-building and skills practice; online for flexibility; weekend timing advantageous • Need to define boundaries, time commitments, and expectations to protect mentor wellbeing •Training, supervision, and quality assurance essential •Integration with healthcare teams and existing patient networks; joint sessions valued •Logistical and structural considerations: awareness, access, and financial/legal support mechanisms

#### Psychosocial challenges and adjustment after transplantation

Participants described substantial psychosocial adjustment demands across the transplant continuum. Although many reported substantial improvements in physical functioning and overall wellbeing, they simultaneously highlighted ongoing vulnerability, physical pain episodes, and fluctuating psychological resilience. Increased awareness of the transplanted organ, persistent fears of graft rejection, and challenges integrating the graft into one's identity were common.

Emotional sequelae included guilt toward living donors, particularly close relatives, and concerns regarding the “burden” placed on family members during recovery. Participants also noted that treatment-related limitations and fatigue could compromise coping abilities during critical phases of recovery. Social strain emerged as a salient factor, with individuals describing isolation tendencies, strained family dynamics, and uncertainty in navigating interpersonal relationships post-transplant.

Vocational reintegration represented both a desired marker of recovery and a challenge, with some participants striving to regain independence and financial stability while others faced physical or psychological barriers. Participants believed peer mentors could meaningfully support emotional processing, normalize post-transplant fears, and offer guidance for reintegration into family, social, and occupational roles.

#### Navigating complex information and the effects of trusted peer experiences

Participants consistently emphasized the need for comprehensible, context-sensitive information across the transplant trajectory (e. g., explaining the purpose and risks of pre-transplant tests, how the waiting list works, and lifestyle). They reported that clinical information was sometimes presented in technical language or lacked sufficient depth regarding real-world experiences, particularly related to immunosuppression, side effects, recovery timelines, and day-to-day management of restrictions.

Patients expressed a desire for early and repeated education regarding dialysis modalities, decision-making around organ acceptance (including marginal organs), donations types, and the organ allocation process. They further advocated for interdisciplinary instruction involving medical, psychological, nutritional, and physical rehabilitation expertise.

Participants highlighted the unique value of experiential knowledge, describing peer interactions as vital for transforming biomedical guidance into actionable self-management strategies. These strategies included adapting medication schedules, implementing lifestyle modifications, and developing coping approaches suited to the demands of everyday life. Peer mentors were viewed as instrumental in demonstrating how medical advice could be realistically applied within personal, social, and practical contexts across the transplant trajectory. Low-threshold mechanisms for peer exchange—such as brochures, online platforms, discussion groups, and family-inclusive sessions—were strongly endorsed. Participants emphasized the need for involving family members early to mitigate misunderstandings related to infection risks, lifestyle restrictions, and emotional burden.

#### Qualifications, roles, and matching of peer mentors

Participants conceptualized peer mentors as credible, empathetic role models with extensive lived experience. Desired characteristics included at least several years of post-transplant experience, emotional stability, and the capacity to communicate with sensitivity, respect autonomy, and maintain professional boundaries. Mentors were expected to possess foundational psychological and communication skills, demonstrate self-reflection, and distinguish personal narratives from generalized guidance. Screening for suitability and psychological readiness was widely supported.

Participants recommended matching based on demographic and experiential characteristics—including age, gender, treatment modality, language, educational background, and geographic proximity—to facilitate rapport and comfort discussing sensitive topics (e.g., fertility, sexuality, body image). They emphasized that mentors should act as supportive companions rather than substitutes for healthcare professionals. Flexibility to reassign mentors in the event of poor fit was considered essential. Some participants also valued the presence of mentors during clinical visits (e.g., in waiting rooms), when requested, as a source of reassurance and emotional support.

#### Barriers and facilitators to engaging in peer support

Participants described several factors that influenced their preferences for how a peer mentoring program in transplantation medicine should be developed and implemented. These included the types of topics to be addressed, preferred delivery formats and timing, and expectations regarding the scope and boundaries of mentor–mentee interactions. Participants identified both barriers to participation, such as unmet informational needs and logistical constraints, and facilitators, including opportunities for trust-building, flexible access, and early integration of peer support.

Participants reported that several topics important to their adjustment and wellbeing were insufficiently addressed during standard pre-transplant education and informed consent discussions. Body image issues and sexual health were frequently mentioned as neglected areas. Although many participants did not personally experience body image concerns, some recognized that visible changes, such as the presence of a shunt, could cause distress for others. Several participants expressed interest in having a mentor who could openly discuss sensitive issues, including pregnancy, sexuality, and intimacy. Occupational challenges following transplantation were also identified as a key area for peer support. Participants wanted guidance on how to disclose their condition to employers, manage potential disability, maintain employment, or return to work after recovery.

Most participants favored in-person meetings for developing trust and fostering a sense of connection. However, they also recognized the benefits of flexible formats. Combining in-person and online options was viewed as optimal to accommodate differing needs and schedules. Online sessions were considered convenient and time-efficient, while certain aspects—such as training in communication or psychological support skills—were seen as better suited to in-person settings. Participants also expressed a preference for weekend-based sessions to enhance accessibility. Joint sessions led by both healthcare professionals and peer mentors were viewed as particularly valuable for integrating experiential and clinical perspectives.

Participants emphasized the importance of introducing peer mentoring early in the treatment process. Most agreed that peer support should be available soon after diagnosis to allow patients to access guidance whenever needed. However, views varied on the ideal duration of mentorship. Some participants felt mentors should be available on an ongoing basis, while others preferred time-limited arrangements to reduce potential burden on mentors and to maintain clear boundaries. Several participants highlighted the need for explicit guidance on how and when to set these boundaries to ensure that both mentors and mentees felt comfortable and supported.

Overall, participants emphasized that an effective peer mentoring program should be comprehensive, flexible, and personalized, addressing unmet informational needs while providing multiple avenues for engagement and support throughout the transplant journey.

## Discussion

This qualitative study examined kidney transplant recipients' (KTRs) perspectives on the content, structure, and delivery of a patient-centered peer-mentoring program. These four key themes emerged to be important when developing and implementing a kidney peer-mentoring program. Our findings highlight that KTRs perceive peer mentoring as a complement to professional transplant care, particularly for addressing emotional, informational, and social challenges that persist throughout the transplant trajectory.

Participants emphasized that living with a kidney transplant is associated with substantial psychological and social adjustment demands. Peer mentors were perceived as uniquely positioned to normalize these experiences through shared understanding and lived expertise—providing authenticity that professional caregivers cannot replicate. The findings underscore that effective transplant support must go beyond biomedical education to include psychosocial coping, self-efficacy, and identity integration, aligning with the broader aims of patient-centered care.

Participants also identified a strong need for clear, comprehensible, and tailored information. Many felt overwhelmed by complex medical communication and found that professional education alone was insufficient to prepare them for post-transplant life. Peer mentors were viewed as potentially credible intermediaries who could translate medical information into accessible, experience-based knowledge and support health literacy. Collectively, these findings illustrate that peer mentoring fulfills a dual role: enhancing psychosocial wellbeing and bridging informational gaps, thereby promoting patient empowerment and engagement across the continuum of transplant care.

Our findings are consistent with prior research demonstrating that peer-based interventions improve psychosocial outcomes, adherence, and self-management among individuals with end-stage kidney disease (ESKD) and other chronic conditions (Dennis, 2003; Taylor et al., 2016; Pomey et al., 2021; Longley et al., 2023). Studies by (Pomey et al. 2021) and (Taylor et al. 2016) similarly found that transplant recipients value peer mentoring for its empathetic, experiential nature and for fostering a sense of belonging and hope. Our findings extend this literature by detailing the specific psychosocial domains (e.g., graft-related anxiety, guilt, vocational reintegration) that patients believe peer mentors should address.

Compared with peer navigation and advocacy models described by (Patzer and Larsen 2019); (Jolly et al. 2015), and others (Myaskovsky et al., 2012), our study provides complementary insights focused on program design rather than outcomes. While navigators in U.S.-based programs primarily facilitate access to transplantation and equity in the evaluation process, our findings emphasize how peer mentors can enhance post-transplant quality of life (QOL), psychosocial adjustment, and self-efficacy. The patient-centered emphasis on emotional and relational components contributes to a more holistic framework for peer involvement in transplant care.

The call for structured mentor training and supervision echoes best-practice recommendations from the Mental Health Commission of Canada (Sunderland et al., 2013) and renal peer-support frameworks (Elliott et al., 2023, 2022; Trasolini et al., 2021; Wood et al., 2022). Participants' preferences for hybrid (in-person and online) formats and interdisciplinary collaboration align with contemporary models promoting flexibility and integration into clinical workflows (Pomey et al., 2021; Bennett et al., 2018). Participants' focus on empathy, communication skills, and boundary setting reinforces the need for professional oversight to safeguard quality and sustainability of peer-delivered support.

Drawing on our findings, several evidence-informed implications can be derived for the design and implementation of a patient-centered peer-mentoring program in kidney transplantation.

First, mentor recruitment and matching should deliberately account for experiential, demographic, and psychosocial congruence (e.g., age, gender, treatment modality, and geographical proximity) to foster trust and relational alignment between mentors and mentees. Participants emphasized that perceived interpersonal compatibility is fundamental to effective engagement and should be supported by mechanisms that allow re-matching when dyadic relationships prove suboptimal.

Second, the establishment of comprehensive, structured training for peer mentors is imperative. Such training should encompass core areas including transplant-related medical knowledge, effective communication strategies, and emotional self-regulation co-facilitated training sessions involving healthcare professionals and experienced peers may enhance interdisciplinary collaboration and program legitimacy.

Third, timing and accessibility emerged as critical determinants of program success. Participants advocated for the early introduction of mentoring opportunities—ideally initiated at the time of diagnosis or transplant listing—to promote emotional readiness and informed decision-making. Offering flexible modalities, such as hybrid and weekend-based sessions, may reduce logistical barriers and better accommodate diverse patient circumstances.

Fourth, the integration of peer mentoring within existing healthcare and self-help networks (e.g., the Austrian self-help association ANÖ and national transplant centers) is essential for program sustainability. Formal institutional recognition, continuous supervision, and potential financial remuneration can reinforce mentor commitment and program quality. Clearly defined role boundaries are required to distinguish peer mentors from healthcare professionals while simultaneously promoting multiprofessional collaboration.

Collectively, these patient-centered recommendations underscore that peer mentoring should not be regarded as an adjunctive service, but rather as an integral psychosocial component of comprehensive, patient-centered transplant care.

Future research should evaluate the feasibility, acceptability, and effectiveness of peer-mentoring interventions guided by the components identified in this study. Quantitative and mixed-methods designs could assess outcomes such as patient-reported quality of life, adherence, and health literacy. Implementation research could determine how best to integrate peer mentoring within multidisciplinary transplant teams and across diverse healthcare systems. Moreover, research should examine peer mentors' own experiences and wellbeing, ensuring that mentorship remains beneficial for both mentors and mentees. Cross-cultural adaptations and comparative studies between healthcare systems would advance generalizability and inform international standards for transplant peer support.

This study's strengths include its national scope, inclusion of participants from multiple Austrian transplant centers, and rigorous adherence to qualitative standards. Conducting in-depth interviews allowed participants to express their lived experiences and preferences freely, ensuring that findings authentically reflect patients' voices. Triangulation of coding by multiple researchers enhanced analytic rigor and credibility.

Several limitations should be noted. Participants were recruited through the Austrian self-help association (ANÖ), which may have led to selection bias toward more engaged or health-literate individuals. Some participants received their transplant many years ago, which may introduce recall bias; however, this long-term perspective also provided valuable insights into sustained challenges and coping strategies. As the peer mentoring framework was conceptualized within the Austrian healthcare context, transferability to other cultural or structural settings may require adaptation. Despite these limitations, our study provides rich and actionable data to inform the development of peer-mentoring interventions in transplant medicine.

## Conclusion

This study provides comprehensive patient-centered recommendations for developing and implementing a kidney peer-mentoring program based on qualitative interviews with patients nationally. This study advances the field by illuminating patients' preferred topics and needs of an effective peer education/mentoring model, and providing insights for developing a theoretical framework for peer support in kidney transplantation. Our findings provide a foundation for designing structured, evidence-informed, and sustainable peer-support interventions that address patients' psychosocial and informational needs across the transplant continuum.

## Data Availability

The raw data supporting the conclusions of this article will be made available by the authors, without undue reservation.
