# Supplementary material for: Development of a patient-centered peer-mentoring program for kidney transplant patients: a qualitative study
Source: Front Psychol. 2025 Nov 28;16:1712921. doi: 10.3389/fpsyg.2025.1712921 (PMC12698399; doi:10.3389/fpsyg.2025.1712921)
Supplement: Supplementary file 1 [file Supplementary_file_1.pdf]

Supplemental appendix

Identified themes, subthemes, and illustrative quotes

Themes	Subthemes	Supporting sample quotes
**Psychosocial challenges and adjustment after transplantation**	Increased physical functioning, reduction of psychosocial distress and overall improved QOL after successful transplant	*"When the kidney arrives, you have 100% quality of life, so I can do sports...I can do everything."*
	Changes in the way of thinking, getting more open-minded	*"With the first kidney, it seemed to me that I tick a little differently. With a piece of different flesh comes a different soul and sometimes I've noticed something about myself where I've thought to myself that I've always remained the same, but something is different."*
	Dealing with physical limitations and pain	*"I got three abscesses in my muscles, which worsened me so massively for three weeks..., so I was almost desperate at times, above all I also had extreme pain."*
	Mental limitations that cause difficulties to keep up with treatment course	*"Before the transplant, you reach your limits, both physically and mentally."*
	Increased awareness for the graft and anxiety of graft loss	*"Hopefully the kidney will last, hopefully it will stay, hopefully it won't be rejected."*
	Physical and mental challenges to cope with treatment and graft acceptance	*"It was a huge shock when the doctor told me that they were children's kidneys and that I would be getting both transplants because they were still so small. There was also a bit of sadness about what must have happened for me to get these kidneys."*
	Challenges in social life pre- and post-transplant	*"In general, it would be very helpful to have more support at home or with children after the transplant, because it is very difficult to manage."*
	Vocational rehabilitation – 'getting back to work'	*"It was important to me that I never lose my independence and that I earn enough money again as quickly as possible to have a good life, but that's not possible for everyone."*
**Navigating complex information and effects of trusted peer experiences**	Get in contact with other patients	*"When it comes to kidney transplants, the association was the thing that helped me the most! Talking to the members on the phone for example."*
	Understandable information – informed consent based on patients' level of knowledge, considering individual needs to be informed	*"But perhaps not in medical language, but in a way that the average consumer can understand, in a simple construction, so to speak."*
	Information about 'real' life before/after transplant (Do's and don'ts, adverse events of transplant)	**Medication management and intensive after care:** *"You could perhaps bring people back to reality by saying that you are still ill, you can live better, but in the background, there are now drugs that have side effects, close-meshed examinations. Such stories are kept quiet and the euphoria of the patient is left, but the reality is then very quickly different."* **Pain after transplant:** *"That it may be said that it is*

	painful until the bladder has expanded again."<br>**Challenges to cope with post-transplant course:** *"Before the transplant, I would have liked to know which symptoms occur one after the other. When do the edemas appear, how many appear, what about the itchy skin, what about sexuality, what about muscle atrophy and fatigue?"*
Low-threshold psychosocial support for patients and donors/family members, facilitating easy access to support;	*"In my opinion, (psychosocial) support, or at least a conversation, would be important for both those affected and their relatives."*
Development of information materials for patients that comprise all relevant details (post- and pre-transplant)	**Contacts:** *"So, if there was a good, standardized brochure (after the transplant), which also contains contact details, contact persons, what you should look out for and how you should live your life etc."*<br>**What you will need for undergoing transplantation:** *"Right from the start, you have to be properly informed about everything that is involved, right up to the transplant or until you are put on the list. All the steps have to be taken, which is why it is very helpful to be properly informed. Also including information about your stay in the clinic and life afterwards."*<br>**FAQs:** *"If there was a website where the most important questions were answered in general terms. You can always add something in some cases. But for example, what's it like when you get on the list and how long you're in hospital and so on."*
Information about the donation (living donation vs. post-mortem transplant) and organ allocation process (Eurotransplant allocation procedure)	**Organ of inferior quality:** *"It was also the case that a nephrologist said to me after the transplant: You already know that you received a kidney that was already somewhat calcified. [...] It was very different between an anonymous donation and a living donation."*<br>**Allocation process:** *"Basically, I would be in favor of the other person accepting the transplant as quickly as possible so that he/she can also deal with the issue. What happens there anyway, what about the donor, the whole background story that happens during the transplant? Where do the organs come from? I would find out more about that, I honestly have to say that I had no idea in the past."*
Interdisciplinary information involving all health care professionals of the transplant field	*"After the transplant I think you need to talk to a dietician, a sports specialist, what sports I could do because I couldn't lift weights. I didn't have all those so maybe that is why the recovery took a lot."*
Creation of transplant networks to support information transfer and exchange of experiences	*"The contact with each other, with fellow patients, with transplant recipients or pre-transplant recipients, the conversations with other patients are even more valuable."*
Peer process could help improving the experiences of	*"Yes, it has helped me in that you can talk about medication and how everything works after the*

patients – ‘make it easier for the patients’	*transplant and simply about some things you're unsure about. For example, if you get a fever or something. I also did something like that during dialysis.”*
Patient advocacy: involving peers in the information process from the beginning, helping to understand medical issues, connecting link between patients and health care professionals	*“That you have a peer at your side who can help you overcome your uncertainties, because they can answer many questions from theory or from their own experience.”*
Get in contact with several peers: multidimensional perspective on the positive and negative effects of getting a transplant	*“I actually find it very good to talk to other people affected. With several and not just one person, simply because everyone has a different story and because everyone may have had a different starting situation.”*
Preparedness and resources of health care professionals to take care of personal patients’ issues, encouraging patients to ask questions	*“I looked at the folders before the transplant, but I was too cowardly to ask questions. I think you should be more proactive in approaching patients who are already on dialysis and are about to have a transplant.”*
Suggestions for improving the information process	**More detailed information:** *“There should be much more information, much more detailed and tailored to the respective situation. Food was also an issue. What you can still eat, what you can no longer eat, there are a lot of differences. Then it's always said that you can't eat this and you can't eat that, while other doctors say, ‘Forget it, you can eat anything now. So more detailed information would be good!”*<br>**More specialized information:** *“We had to do a lot on our own initiative. Of course you were informed at the clinic, but you have to write and call various hospitals, outpatient clinics and doctors to get a bit further. So that you know, for example, what it means to transplant against the blood group, what tests do you actually need for this, when is something ruled out or is everything simply always possible? There is actually no support at all.”*
Involvement of family and friends into the information process	**Involvement of the donor:** *“Living donation is very different from post-mortem donation, because you have both partners on board and can talk to both of them, so you accompany both of them.”*<br>**Family and friends:** *“Friends and family should be very closely involved, because unfortunately the patient often makes the mistake of covering up many things. And so those around them are very surprised when the transplant is suddenly due and only then do they realize how much they are actually suffering. I always covered this up very skillfully, which is why my family was very surprised. There were also a lot of misunderstandings in the family, where people would say, 'Why isn't he here today? Well, I skillfully covered up my situation in everyday life, but I still had my bad*

		days. That's why you should definitely involve and inform the family, because many don't even know what a transplant is, many don't know what it means to have to take immunosuppression for a lifetime. These are simply topics that the people around them are not well informed about, which then causes many problems in everyday life because the transplant recipient has to be looked after. There have been a few situations where relatives have come with their children who had coughs and colds. They simply weren't aware of the risks involved for me."
**Role/Tasks of peer mentors and their qualifications**	Open-minded attitude of peer mentors, optimistic basic attitude	*"The most important thing is a positive attitude."*
	Empathic attitude and communication at eye level	*"That the peer mentor is empathetic, that they also understand what it means to communicate on an equal footing."*
	Long-term personal experiences after transplant – higher experience value	*"I think that this should be a person who has been transplanted for a longer period of time. So, I'd say five years plus, maybe even more.It doesn't matter how many kidneys you've had, it's simply about the experience a person gains over the years. If you were only transplanted two years ago, you may not have been on vacation yet or you may not have experienced a situation at work where another person asked a stupid question. The longer you have been transplanted, the more experience you have with various situations in life."*
	Evolved knowledge about transplantation to inform and advise patients	**Knowledge about essential medication questions:** *"Of course, I would like peer mentors to be quite well informed when it comes to technical terms or what creatinine is and to be able to answer such questions."* **Knowledge about the transplant course in detail:** *"I could well imagine talking to someone who has already had the transplant. The person can then give you good tips on how the whole thing will turn out and that you can look forward to the whole thing positively."*
	Focus on medical facts, no impart half-knowledge	*"To have the self-conception that if I don't know something, then I admit it, either I inform myself afterwards and provide the information, but not to make the mistake of then saying something."*
	Basic psychological skills to get in contact with others – exclusion of patients with mental disorders like schizophrenia, dementia, high level of personal suffering, cognitive impairment, etc.	*"A certain amount of basic knowledge, not medical knowledge, but also in conducting conversations, i.e. being communicative, being able to listen, etc. These are a few criteria that are important."*
	Successfully dealing with one' own history – sensitivity to	*"Not just reciting their own story, so to speak, but simply being able to distinguish between objectivity*

differentiate between subjective and objective issues	*and subjectivity, that human bodies are very different and opinions on medical therapies can be very different."*
Not competing, who suffers more, which transplant was more challenging - we are all in the same boat	*"It also always requires a great deal of empathy. Very importantly, the other person is the focus. It's not about telling my story, but that the other person gets through it well and is advised!*<br>*It's not psychotherapy for the person who is helping, it's support for the other person."*
Open-minded discussion about a patient's choices, respect for patients' autonomy and impartial advice	*"A patient, with his diagnoses and the whole thing pretty hopeless and he decided to stop the therapy and die. [...] It was a huge challenge for me [...] just to listen, not to confirm that what he was doing was good, but to respect his wishes, his concerns and that he could talk in a neutral way with someone who wasn't trying to influence him or exert any emotional pressure."*
Common similarities between peer mentors and patients	**Language:** *"My problem is, I cannot speak fluent German like most people in Austria."*<br>**Age:** *"Age, of course, because a 20-year-old and a 60-year-old simply have different views."*<br>**Gender:** *"The most important thing, in my opinion, is that they are same-sex interviewees, because then the questions are also asked differently."*<br>**Similar course of disease:** *"It's just positive when you hear from someone else that they had the same problem and that it's not so bad."*<br>**Profession and level of education:** *"Well-educated with well-educated or well-educated with less well-educated, this should perhaps be taken into account."*
Role model function – peer mentors present a sample of how life could look like after transplantation	**Appearance of the peer mentors:** *"If the peer mentor is unfriendly or comes across as neglected or incompetent, he/she can then tell a lot and leaves quickly. So, I think there should already be a certain demeanor there."*<br>**Presenting a healthy lifestyle:** *"It also makes you feel optimistic about the future when you meet transplant recipients who look healthy at first glance and who appear to be anchored in life and able to live everything you could wish for."*<br>**Adherence to treatment plans:** *"Something similar to you happened to me and I'm fine now. I looked after myself during dialysis and I listened to the doctors [...] You just have prospects again."*
'Peer mentors should be friends' – low-threshold information exchange and discussing everyday issues	*"I would say, first and foremost, on a friendly basis, that you have trust, because then it's easier to talk."*
Focus on the patients' level, peer mentors must not take over the tasks of health care	**Focus on patient level:** *"Again, contact those affected, because if you have questions or concerns, these are sometimes things that don't necessarily have to be answered by a doctor. So, if someone has had a*

	professionals/demarcation of health care professionals	*transplant for a long time, these are often questions that are emotional."*<br>**Demarcation of health care professionals:** *"I think it's important to delineate where your options go and where another profession is responsible."*
	Near to home contacts	*"Perhaps it would also be important that the distance between peer mentor and patient is considered. So, for example a person from Vorarlberg does not have to talk to a person from Vienna. [...] Because I think the personal aspect would certainly be more important than a phone call."*
	Installation of a pool of peer mentors and patients could select one for themselves	*"Maybe it would be good in this case, if you have a pool of peer mentors, to tell the person concerned who he/she would like to choose for themselves."*
	Trained self-help-work and duties of peer mentors	**Trained self-help-work:** *"I can see with our trained peers (the organization) that these people have completely different starting points than people who have not undergone this training. You can reach the patient much better, and you can tackle problems and thoughts much better where a non-trained person cannot. That's why I'm very convinced of the role of the peer."*<br>**Duties:** *"Yes, like in an institution, the peer mentor could visit the dialysis stations, make contact with people and look after them, arrange meetings and also confer with the new media."*
	Peer accompaniment and organizational support	**Accompaniment to medical interventions at the patients request:** *"The question is whether a peer can also go along as a companion in some form, so I think perhaps not directly at the doctor-patient consultation, but that he is there in the waiting area for reassurance before the appointment starts. Perhaps some patients need support before a certain examination."*<br>**Organizational support:** *"That the other person says, for example, look, you're getting the package with the medication now and your first appointment is on such and such date at the hospital, no matter where. That would of course be the most important thing.*<br>*Of course, I went to my GP, who doesn't know where I have to go for my next check-up either. She just prescribed the medication and that was it."*
	Advantages of being a peer mentor: deepening own knowledge about transplant, altruistic experience/helping others	*"On the one hand, to expand and deepen your own knowledge and, on the other hand, to support and accompany people who are already on the same path as you and to be available as a discussion partner."*
**Barriers and facilitators to engaging in peer support**	Basic medical knowledge and transplant related knowledge	*"The person should be familiar with and know the practical circumstances of kidney transplant and dialysis patients, the dialysis process, etc. What the main problems are for the respective patient and what the issues are in the respective group of affected patients."*

	**Insulin and blood pressure:** *"Side effects of immunosuppression, diuretics, what's going on with your blood pressure, will it get better, why do you have to inject insulin when you didn't have to before, stories like that, in other words, more of medical nature."*<br>**Risk of cancer & NO-GOs:** *"After the transplantation, there are several things you should pay attention to, such as going to the dermatologist regularly and the information on what to look out for. Basically, you're fit again, but you shouldn't drink grapefruit juice or use St. John's wort oil, for example. I actually only learned all this at the association, I wasn't told enough about it in hospital."*<br>**Transplant process:** *"The complete process of a transplantation from start to finish, beginning with the call and ending with the return to work. And in between, the entire spectrum of what happens and what needs to be done."*
Dialysis and preparing for potential transplant	*"The crucial point is when people find out that their kidney is broken or will be broken. The second most important point is that you then help people make the decision to go on dialysis. Here in particular, it is very important to promote peritoneal dialysis, which can be done at home. It is cheaper and less stressful, but of course you have to take responsibility for it because you have to do it yourself. Hemodialysis is done by someone else."*
Information about a healthy lifestyle	**Smoking and consumption of alcohol:** *"It is certainly important to provide information about health, to talk to people and to tell them how important it is to exercise after the transplantation and to improve their lifestyle a bit more if possible. There are also people who previously drank alcohol or smoked, so it is important to help them get on a healthier path."*<br>**Hygiene, Nutrition and Physical training:** *"One topic that is always of interest is nutrition and hygiene. We always try to convey this in our patient magazine, that it is important to have a reasonably healthy lifestyle, with physical activity and exercise and a good diet, and that fast food is not part of this."*
Taking care of emotional issues	**Coping with anxiety:** *"For me personally, the psychological aspects are very, very important. Fighting fears, tackling fears in the right way and, above all, if you have fears, you find yourself in a dead end and that's where it makes sense to lead the transplant recipient out of it using certain approaches and to work out certain aspects and goals together and get there together."*<br>**Guilt towards the donor:** *"I was more concerned that my girlfriend had her body cut open to remove a healthy organ. I was worried, and that would have made me sad, if the organ hadn't worked for me."*

Communication skills and basic psychological training	*"Tips for communication, i.e. how do I deal with difficult characters, how can I build up a conversation if the concerned person is very unapproachable, so basic tips, similar to what a trainer should have. It's important to be able to communicate with different types of people, to be able to say no and to be able and allowed to say negative things."*
Involving family members: how to be cooperative with family members	*"How do you deal with family members? Do you involve them or is it just a one- on-one conversation? What if someone says 'I want my husband, my wife, my children to be there'?"*
Pre- and post -transplant body image issues (in case of need, not for all patients)	*"To be honest the shunt grows bigger with 3 and a half years of therapy, so I cannot wear a T-shirt. Someone my age with a shunt or a person of old age with a shunt, there is a difference, how people look at you and how comfortable you feel, when it comes to physical appearance."*
Sexual life and pregnancy	*"Sexuality, which also caused me problems, is not addressed directly."*
Information about non-medical, transplant-related issues	**Work:** *"Social issues that affect patients should also be addressed. Many patients are affected by occupational disability or partial occupational disability, and other social issues are also important to discuss. So, this should also be addressed."*<br>**Social Security:** *"The disability status classifications from the Federal Social Welfare Office, and the parking permit for the disabled. Dialysis patients are still slipping through the cracks at the moment. This is actually set far too low. Transplantation, for example, is given a disability rating of 50%. That's why an improvement here would be really desirable."*<br>**Subsidies:** *"Advice regarding the financial situation and options would be useful. For example, I wasn't aware for years that I could have applied for housing subsidies."*
Well-considered selection of potential peer mentors	*"I don't think that every person is ideally suited to this role. You need a sensitivity for other people and a lot of empathy. Above all, you need people who can connect well with the other person and they should be healthy transplant recipients, not people who are just about to undergo transplantation."*
Integration of the peer-mentoring program in existing networks (self-help associations and medical care course), collaboration with self-help associations	*"How the peer mentors act alongside the self-help groups, so to speak, or whether they perhaps act with the self-help groups or are part of the self-help groups. [...] In this landscape around the patient, it is very important that the doctors and the self-help groups get on well together and when the peer mentors join in, they also have to fit into the system somehow, so they can't act alone."*
Finance resources and legal questions regarding the peer	*"The question is, what status or what legal safeguards and opportunities do these people (peer mentors)*

education course, financial support to acknowledge the work of peers	*receive?"* *"It always depends on whether it's paid or not, that's also the question."*
Social media and online applications (e.g. website of the peer network), advertising the peer-mentoring program	*"It would be best if there was some kind of platform where people like me could register. I could then provide information about living donation against your own blood group. People could then get in touch and ask questions, if it's not already available from the medical side!"*
Decentralized course model based on mixture of lessons attended in person and online	*"Three hours online would be best, because that gives you a lot of flexibility, but just online isn't good either. Some kind of hybrid story would certainly be ideal."*
Weekend course model vs. predetermined appointments	*"Based on my gut feeling, I would say a weekend course. It always depends on whether there are people in employment or not, but I think the weekend course would suit everyone. For employees, the weekend course might be better."* *"In my rotational service, it would of course be good if this kind of training could take place in a compact way. Possibly also via distance learning or Zoom, because people's everyday lives are simply very limited. But I think the more flexible the whole program is, the better."*
Organization of low-threshold lectures by health care professionals (physicians, mental health care professionals, and caregivers) and peer mentors	*"I would say that personal meetings should be pushed more, also with regard to doctors. We should make sure that the doctors, who carry out dialysis or general preparation for transplantation in the respective hospital, hold meetings and lectures at least once a year. That you can talk to each other, but also to the doctors, that would be the most important thing."*
A pool of peer mentors that would help to identify peer-patients-matches and to make new assignments in case of mismatches	*"There are topics where the chemistry isn't right. But then the peer mentor must be ready to say, I'll send you someone else, we won't get on and someone else should do it."*
Ongoing supervision and intervision of peer mentors, quality control	*"What's not quite clear to me yet [...] is whether they will also have something like accompanying supervision later on or whether there's only one training course and then they can do whatever they want, how it's organized, how there's some form of quality assurance, whether they exchange information with each other, things like that."*
Timeline that clarifies peer mentor involvement in the treatment course	*"I think that this kind of contact should be made as early as possible. If someone knows that they will need dialysis, to provide the relevant information as soon as possible."*
Defining a regulation of time requirements to work as a peer mentor	*"As often as the patient wants and also at any time, the peer educator says 'Call me if you have a problem'."* *"That might also be a topic for such training or courses, how do you establish boundaries?"*

Modalities that define how peer mentors and patients communicate and interact (online vs. face-to-face)	*"Personal contact is better, but it is also possible online. It depends on the situation, i.e. the risk of infection and so on. Both options must be possible, but personal contact is certainly better."*<br>*"I think it should be a personal contact. But you should be able to choose that individually, because one person prefers it this way, another that way.*<br>*A young person probably prefers a video conference, while an older person needs more personal contact."*
Psychosocial support in form of group offerings	*"I see it very creatively, I think anything is possible, from one-to-one meetings to group processes."*
Reasons not to become a peer mentor	**Age:** *"I could imagine going through this kind of training, but I have to admit that I'm too old. Not my condition, not my willingness and not my head are the problem, but let's just say that once you're over 80, life is different in many ways."*<br>**Time:** *"If the training becomes too intensive and too time-consuming."*<br>**Health:** *"Unfortunately, I'm just not strong enough at the moment to take part in a program like this, but when I'm stronger and fitter again at some point, I would be interested in a program like this."*<br>**Privacy:** *"Maybe you have the transplant and you don't want to tell everyone out there, maybe that is an argument, because many people want to keep things private."*

Note: Only illustrative quotes were provided to ensure the table remains concise.
